# Conventional Biological versus Sutureless Aortic Valve Prostheses in Combined Aortic and Mitral Valve Replacement

**DOI:** 10.3390/life13030737

**Published:** 2023-03-09

**Authors:** Alina Zubarevich, Marcin Szczechowicz, Arian Arjomandi Rad, Lukman Amanov, Arjang Ruhparwar, Alexander Weymann

**Affiliations:** 1 Department of Thoracic and Cardiovascular Surgery, West German Heart and Vascular Center, University of Duisburg-Essen, 45147 Essen, Germany; 2 Clinical Academic Graduate School, University of Oxford, Oxford OX1 2JD, UK

**Keywords:** Perceval, combined procedures, SAVR, SMVR, SU-AVR, multivalve disease

## Abstract

Background: Sutureless aortic valve prostheses have proven to provide a significant decrease in procedural, cardiopulmonary bypass and cross-clamp time, leading to a significant reduction in mortality risk in elderly high-risk cohorts. In this study, we sought to review our institutional experience on the sutureless aortic valve replacement (SU-AVR) and the concomitant mitral valve replacement (SMVR), comparing the combined conventional surgical aortic valve replacement (SAVR) with SMVR. Methods and Material: Between March 2018 and July 2022, 114 consecutive patients underwent a combined aortic and mitral valve replacement at our institution. We stratified the patients according to the operative procedures into two groups and matched them 1:2: Group 1 underwent a combined conventional SAVR and SMVR (n = 46), and Group 2 included combined SU-AVR with Perceval prosthesis and SMVR (n = 23). Results: No significant differences in the preoperative characteristics were present. SU-AVR combined with SMVR demonstrated excellent haemodynamic performance, comparable to that of SAVR plus SMVR, with median postoperative gradients over the aortic valve of 4 mmHg (IQR 3.0–4.0) in Group 1 and 4 mmHg (IQR 3.0–4.0) in Group 2 (*p* = 0.67). There was no significant difference in the occurrence of postoperative major adverse events such as death, stroke, myocardial infarction and kidney failure between the groups. There was also no significant difference in the permanent pacemaker implantation rate, paravalvular leakage or valve dislocation. We also could not detect any significant difference in postoperative mortality between the groups. Conclusions: SU-AVR has proven to be a reliable alternative to conventional valve prostheses in patients with multivalve disease undergoing combined aortic and mitral valve replacement, offering shorter procedural time and outstanding hemodynamic performance compared to the conventional surgical method.

## 1. Introduction

Aortic stenosis (AS) represents the most prevalent valvular disease in adults, affecting between 2 and 7% of the population over the age of 65 [1]. Aortic valve replacement (AVR) remains the gold standard treatment for patients with severe symptomatic aortic stenosis, having been shown to provide excellent long-term mortality and morbidity results. Global epidemiological shifts have presented an increasingly elderly and generally more comorbid patient population to cardiac surgeons, which is also prone to an elevated risk of multivalve disease. Moderate mitral valve regurgitation in patients undergoing AVR has been illustrated to constitute an independent predictor factor for mortality. Mitral valve and tricuspid valve surgery, in conjunction with AVR, makes up around 11% of all operations performed by cardiac surgeons, thus constituting a significant proportion of the operative volume [2]. Nevertheless, it must be noted that multivalve procedures have been shown to carry a significantly increased risk of mortality and morbidity, underlining the need for the identification and adoption of optimal treatment strategies.

Recently, transcatheter aortic valve implantation (TAVI) and sutureless aortic valve replacement (SU-AVR) have emerged as alternative treatment strategies for patients with AS deemed high-risk. Despite the rapid expansion of isolated TAVI indications, surgical aortic valve replacement (SAVR) remains the treatment of choice for patients presenting with multivalvular heart disease [3]. Nevertheless, in most cases, patients initially present with significant comorbidities, and thus tend to carry a high operative risk [4]. Lately, a rise in the reports of experiences with transcatheter double valve interventions has been witnessed, but the data on this subject remain scarce and inconclusive, with the depicted cases being mostly high-risk interventions on predominantly inoperable high-risk patients [5].

SU-AVR has demonstrated excellent results both in patients presenting with aortic valve stenosis and in other indications such as pure aortic regurgitation, aortic valve endocarditis and even in multivalve procedures [4,6,7,8,9]. In all these studies, sutureless aortic valve prosthesis (Perceval S, Corcym, Saluggia, Italy) has been shown to provide excellent hemodynamic performance, with low transvalvular gradients at short- and long-term, as well as an easy implantation technique allowing a significant reduction in operating and cross-clamp time. Furthermore, a recent meta-analysis comparing TAVI with SU-AVR demonstrated that SU-AVR is able to provide significantly higher survival at 1 and 2 years, as well as lower rates of paravalvular leakage (PVL) [10]. When PVL is taken into consideration, it must be noted that the surgical implantation of SU-AVR includes extensive decalcification of the root and excision of the aortic valve leaflets, which are not part of TAVI implantation. Moreover, the flexible design of the sutureless valves allows for an adaption of the valve geometry in line with physiological movements of the aortic root. Despite the feared interaction between mitral and aortic valve prostheses at the level of the aorto-mitral continuity, we were able to illustrate the feasibility of the implementation of sutureless aortic valve prostheses for multivalve procedures in our previous reports [4].

Although SU-AVR might be able to provide advantages when compared to standard SAVR, especially in terms of operative times translating into clinical results, the debate about the optimal valve choice remains open with further comparative data needed. In line with our recent research evaluating novel surgical applications of sutureless aortic valve prostheses, we sought to review our institutional experience on SU-AVR in patients undergoing concomitant aortic and mitral valve replacement, comparing it to the conventional combined SAVR and mitral valve replacement (SMVR).

## 2. Methods and Materials

### 2.1. Study Design and Populations

Between March 2018 and July 2022, 114 consecutive patients underwent a combined surgical aortic and mitral valve replacement at our institution. We analyzed and compared the postoperative outcomes and complications of patients undergoing a combined SAVR plus SMVR with those receiving SMVR plus SU-AVR using the Perceval S aortic valve prosthesis (Corcym, Saluggia, Italy). Patients were included if they required combined aortic and mitral valve replacement for any indication. Patients who underwent a mitral valve repair were excluded from the study. We also excluded the patients who received a mechanical aortic valve prosthesis. The choice of the valve prostheses was made by the operating surgeon depending on their preference. All implantations of sutureless prostheses were performed by senior surgeons who have completed the learning curve.

Our Heart Team, made up of experts from various disciplines, reviewed all patients. We evaluated the functioning of the implanted valve prosthesis through postoperative echocardiography both at discharge and during follow-up. Prospective data were collected for our institutional database, including patient demographics, clinical characteristics, lab results, echocardiographic data, hemodynamic parameters, intraoperative variables and postoperative outcomes. The study adhered to the 2013 revised Declaration of Helsinki and received approval from our institutional ethics board (approval #21-10350-BO), waiving individual patient consent. Patients provided signed informed consent for follow-up visits at the hospital.

### 2.2. Study Groups

We stratified the patients according to the operative procedures into two groups. Group 1 included patients who underwent a combined conventional SAVR and SMVR (n = 46), and Group 2 included patients receiving combined SU-AVR with Perceval S prosthesis and SMVR (n = 23). The choice of the aortic valve prosthesis was made at the surgeons’ preference and prosthesis availability at the institution. The patients were matched based on the following preoperative parameters: gender, age, the urgency of the procedure, infective endocarditis, pulmonary hypertension, diabetes mellitus, chronic obstructive pulmonary disease, coronary arterial disease, history of PCI, atrial fibrillation, EuroSCORE II, serum creatinine, ejection fraction, echocardiographic valve evaluation and concomitant procedures, resulting in 69 matched patients 1:2 which have been analyzed. We used a nearest neighbor propensity score matching 2:1 without replacement with caliper = 0.2.

### 2.3. Operative Techniques

The median sternotomy was performed to access the chest. During the procedure, the heart was maintained in a normothermic state of cardiac arrest. The cardiopulmonary bypass (CPB) was initiated with the direct cannulation of the ascending aorta and right atrium, except in cases where bicaval cannulation was required for procedures involving the tricuspid valve. Custodiol-HTK, a product manufactured by Köhler Chemie GmbH in Bensheim, Germany, was administered either through the aortic route or directly into the coronary ostia in cases of severe aortic regurgitation. The native aortic valve was then removed in its entirety through a high transverse aortotomy after being decalcified.

For mitral valve replacement, the valve was exposed through left atriotomy in the Waterson’s groove. Care was taken to ensure proper positioning of the valve prosthesis struts to avoid obstructing the left ventricular outflow tract (LVOT) [4]. One of the struts was placed halfway between the medial and lateral fibrous trigons of the mitral valve annulus and away from the aorto-mitral continuity to prevent LVOT compromise.

The Perceval S sutureless aortic valve was implanted following the positioning of three 4.0-prolene guiding sutures in the middle of each nadir. The implantation was carried out using the Snugger technique as previously described [7].

In cases where conventional sutured aortic valve prostheses were used, they were sized and implanted in a standard manner. The aortotomy was closed using a 4.0-prolene double-layered suture. After the performance of the prosthetic valves was evaluated and any air was removed, the CPB support was discontinued. The anticoagulation was completely reversed, and the chest was closed with steel wires in a conventional manner after ensuring hemostasis was secured.

### 2.4. Concomitant Procedures

In patients undergoing coronary artery bypass surgery (CABG), the left internal thoracic artery was harvested using either a pedicled or skeletonized technique, as determined by the surgeon. Upon initiation of cardioplegic arrest, the target coronary vessels were identified, and the distal coronary anastomoses were carried out in a standard manner. Proximal coronary anastomoses, if necessary, were performed during cardiac arrest without additional clamping of the aorta to avoid damaging the sutureless prosthesis. In the case of a conventional sutured aortic valve, the cross-clamp was released before performing the proximal anastomoses.

The tricuspid valve annuloplasty was performed using a Medtronic Duran AnCore Band on the beating heart during total cardiopulmonary bypass, prior to administering the cardioplegic solution. The left atrial appendage was closed on the beating heart using the AtriClip device (AtriCure, Mason, OH, USA).

### 2.5. Perceval Sizing

The proper sizing of the sutureless prosthesis is essential after the completion of the mitral valve procedure to prevent interference with the prostheses at the aorto-mitral continuity, as previously reported [4]. It should be emphasized that even with proper implantation, the sutureless prosthesis may still cause partial restriction of the left ventricular outflow tract (LVOT). Hence, it is imperative to measure the size of the sutureless prosthesis after implantation of the mitral valve, as the pre-implantation measurements might not accurately reflect the fitting post-implantation.

### 2.6. Outcomes and Definitions

The primary endpoints were 30-day, 6-month and 1-year mortality, as well as mortality at follow-up. The secondary endpoint was the development of any complications according to the Mitral and Aortic Valve Academic Research Consortium (MVARC and VARC) [11,12].

### 2.7. Statistical Analysis

The statistical analysis, including regression analysis, was carried out using IBM SPSS version 27 (IBM Corp., Chicago, IL, USA) and R software version 3.4.3 (R Foundation for Statistical Computing, Vienna, Austria). The normality of the data was assessed using the Shapiro–Wilk test. Given the limited sample size, continuous variables were presented as medians (interquartile range, IQR). Categorical variables were presented as frequencies and percentages, and their distributions were compared using either the Chi-Square Test or the Fisher Exact Test, depending on the normality assumptions. The comparison of continuous variables between groups was performed using either the t-test for normally distributed data or the Mann–Whitney test for non-normally distributed data. A *p*-value of less than 0.05 was considered to be statistically significant. The mid-term mortality was calculated, and survival curves were plotted using the Kaplan–Meier method, and the cumulative survivals of the two methods were compared using the log rank test.

## 3. Results

### 3.1. Baseline Characteristics

The median age of the patients was 71.5 (IQR 64.0–77.0) years (Table 1). All patients presented with symptomatic moderate-to-severe multivalve heart disease, carrying an intermediate surgical risk as portrayed by a median EuroSCORE II of 5.9% (IQR 3.2–10.6). Both study groups did not significantly differ in terms of preoperative characteristics and were therefore well comparable after 1:2 matching.

### 3.2. Intraoperative Data

Out of all the patients, 36 (29.5%) underwent an urgent procedure and ten patients (8.2%) underwent an emergency operation (Table 1). There was no significant difference between the two groups regarding the urgency of the procedure. A large portion of patients in each group (43.5%) underwent a concomitant procedure (n = 20 in Group 1 vs. n = 10 in Group 2; *p* = 1) and Group 2 patients showed significantly shorter operating and CPB times (Table 2). Both surgical methods provided satisfying transvalvular gradients in the immediate postoperative period, over the mitral prosthesis (median MV-MPG 4 mmHg (IQR 3.0–4.0) in Group 1 vs. 4 mmHg (IQR 3.0–4.0) in Group 2; *p* = 0.67) and aortic valve prostheses (median AV-MPG 6.0 mmHg (IQR 5.0–6.0) in Group 1 vs. 6.0 (IQR 6.0–7.0) mmHg in Group 2; *p* = 0.6). There were no cases of intraoperative valve prosthesis dislocation in either group.

### 3.3. Postoperative Complications

There was no significant difference in major adverse events, including stroke, myocardial infarction, new onset dialysis, pacemaker implantation or LVOT obstruction, at follow-up between the two groups (Table 3). Additionally, the median postoperative pressure gradient at follow-up was similar between the groups (median AV-MPG in Group 1 5.0 mmHg (IQR 4.0–6.75) vs. 6.0mmHg (IQR 0–6.0) in Group 2, *p* = 0.55; median MV-MPG in Group 1 3.5 mmHg (IQR 2.0–4-0) vs. 4.0 mmHg (IQR 0–4.0) in Group 2, *p* = 0.8).

### 3.4. Survival

**Figure 1:**
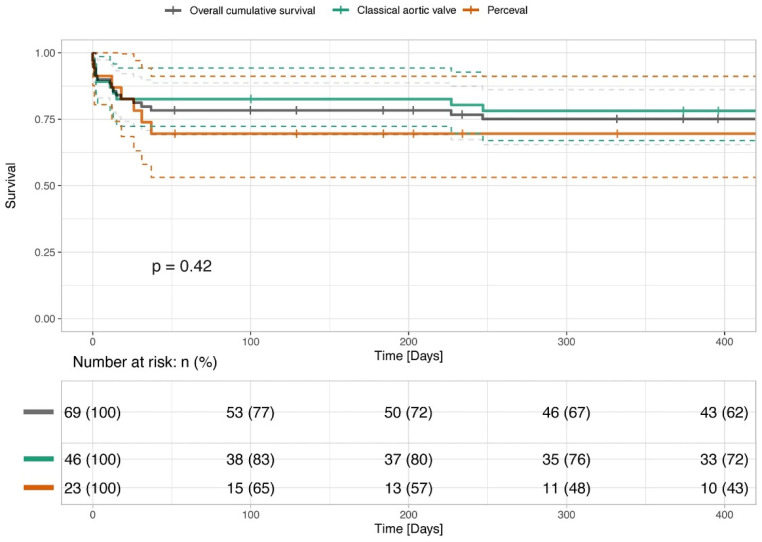
Survival of patients undergoing conventional combined SAVR and SMVR and combined SU-AVR and SMVR.

The median follow-up time was 1.65 years (IQR 0.44–3.35) (Table 4). The 30-day, 6-month and 1-year overall mortality rates did not significantly differ between the study groups as presented in Figure 1, *p* = 0.42. None of the patients in the whole cohort presented with paravalvular leakage. There was no difference in the rate of postoperative aortic valve regurgitation between the groups.

The figure presents the survival of patients undergoing combined SAVR and SMVR and combined SU-AVR and SMVR, presented with Kaplan–Meier Curves. The survival rates have been analyzed and compared with the log rank test and show no statistical difference (*p* > 0.05).

## 4. Discussion

In the following study, 123 intermediate-risk patients presenting with multivalvular (aortic and mitral) heart disease were either treated by combined conventional surgical aortic and mitral valve replacement or a combined sutureless aortic valve replacement and mitral valve replacement.

The findings of this study offer valuable insights:

1. Both surgical techniques showed high procedural success rates and optimal hemodynamics; however, SU-AVR had a significantly shorter operative time.
2. The hemodynamic performance between the groups was not significantly different, with both techniques providing low transvalvular gradients after follow-up.
3. There was no significant variation in the incidence of major adverse events and permanent pacemaker implantations between the groups.
4. No significant difference was observed in the mortality rates at 30 days, 6 months, and 1 year among the groups.
5. The combination of SU-AVR and traditional mitral valve replacement presents a practical alternative to conventional surgical procedures, as it can be performed with ease and has complication rates similar to those of conventional valves.

Patients presenting with combined aortic and mitral valvular heart disease requiring a surgical correction often present with multiple comorbidities and therefore carry a high operative risk. In the German Heart Surgery Report 2001, patients undergoing a combined SAVR and mitral valve repair presented with a 30-day mortality of 5.8%. As soon as the mitral valve needed to be replaced, the 30-day mortality drastically increased up to 15.3% [13]. Considering that rheumatic heart disease is rather uncommon in the Western world, we are looking at a cohort of elderly patients with at least an intermediate surgical risk undergoing a combined valve procedure. Moreover, our relatively high mortality and complications rates could be explained by the fact that in our center the rate of successful mitral valve repair is exceptionally high, therefore those patients undergoing a mitral valve replacement represent a group where the mitral valve is destructed beyond repair, thus carrying the highest operative risk [14,15].

The role of procedural, CPB and cross-clamp time as independent mortality predictors in cardiac surgery is indisputable and has been previously described in multiple clinical trials [16,17]. Ranucci et al. report an increase in risk of 1.4% per 1 additional minute of cross-clamp time [18]. The PERSIST-AVR trial demonstrated the superiority of sutureless aortic valve prostheses compared to conventional SAVR in isolated and combined procedures [19]. Indeed, sutureless aortic valve prostheses provide the advantage of reducing the procedural, CPB and cross-clamp times, consequently reducing the patients’ mortality and postoperative morbidity. In our cohort, we have clearly shown this significant reduction in the procedural time in the sutureless group when compared to the standard surgical group. Although CPB time has not quite reached the significance threshold, there is a tendency towards its reduction. The same findings were reached in the study by Lloyd et al., in which transcatheter, sutureless and conventional aortic valve prostheses were compared [20]. In our cohort, we observed a higher rate of exploration for bleeding in patients undergoing conventional surgical procedures (17.4% in Group 1 vs. 4.3% in Group 2, *p* = 0.25). This could be explained by the positive correlation between the procedural time and the occurrence of bleeding complications after cardiothoracic surgical procedures [21].

Transvalvular gradients are known to be of great importance in light of long-term prosthesis patency and its hemodynamic performance. The Perceval S prosthesis has shown to provide excellent hemodynamics in isolated and combined aortic valve procedures at short, mid and long term, which are comparable to conventional SAVR [22,23,24,25,26]. Similarly, in this study we also observed a satisfying hemodynamic performance of the sutureless prostheses in combined aortic and mitral valve replacement compared to the conventional surgical method.

In early reports of smaller cohorts with sutureless prostheses, there has been a misleading perception that Perceval S protheses tend to cause a higher rate of postoperative pacemaker implantations when compared to conventional aortic valve prostheses [27]. These earlier findings have been overturned by multiple larger trials, including multicenter studies which highlighted a correlation between the surgeon’s SU-AVR implantation learning curve and the pacemaker implantation rate [22,24,25,28]. Indeed, a significant reduction in pacemaker implantation rates has been reported by high-volume centers [22,24,28,29]. Due to the anatomical relations between the position of the conductive tissue and both valve prostheses, combined aortic and mitral valve replacement inherently carries a higher risk of permanent pacemaker implantation when compared to isolated aortic valve replacement. Nevertheless, in our cohort we not only observed a low pacemaker implantation rate of 4.3% in the sutureless group, but this rate was also comparable between the two groups. Our findings are in line with both the previously published results (even in isolated aortic valve procedures) and our own experience with sutureless prostheses in multivalve procedures [4,28,30].

Paravalvular leakage (PVL) is a concerning issue after surgical valve replacement, especially in the context of combined multivalvular heart disease, putting the patients at risk of prosthesis endocarditis [31,32]. In the growing era of TAVI, we somehow got used to accepting mild paravalvular leakage in high-risk patients undergoing this procedure. However, to some extent the same tendency migrated into the low- and mid-risk patients, who, according to the current guidelines, are now also eligible for TAVI [3,33]. A recent meta-analysis of matched studies comparing outcomes of TAVI with SU-AVR demonstrated that SU-AVR has a significantly lower rate of PVL (OR = 0.06; 95% CI: 0.03–0.12, *p* < 0.01) and of 2-year mortality (OR = 4.62; 95% CI: 2.62–8.12, *p* < 0.01) [10]. In a large multicenter European study of over 700 patients, Shrestha et al. report an early postoperative PVL rate of 1.4% in patients undergoing Perceval S implantation [22]. This PVL rate is comparable to both the reported PVL rates after conventional SAVR and the results of our current study, which is even more significant considering that in our case the patients underwent a combined valvular procedure [34]. In this study, we could not show any difference in the PVL rate between the sutureless and the conventional surgical group, although Brett at al. reported higher rates of PVL after surgical combined aortic and mitral valve replacement [31].

In this present study the patients presented with a median EUROScore II of 5.9% (IQR 3.2–10.6), placing them into the intermediate-risk group. As we have already stated, the surgical combined aortic and mitral valve replacement in Germany carries a 15.3% mortality risk [13]. In our cohort, the mortality rates and the occurrence of severe postoperative complications, including myocardial infarction, stroke or kidney failure, were not significantly different between the groups, which again emphasizes the feasibility of the sutureless prostheses implementation in a comparable cohort. We also did not face any technical differences, such as prosthesis dislocation or left ventricular tract obstruction, in the sutureless group. One important aspect to consider, which has been mentioned in multiple studies exploring the use of Perceval with concomitant mitral valve surgery, is the aorto-mitral distance (AMD) assessment. Measurement of the AMD enables safe deployment of the sutereless prosthesis, avoiding prothesis interference and supra-annular mispositioning. In general, an AMD of 5 mm has been considered as a safe measurement when considering the deployment of SU-AVR with concomitant mitral valve prostheses [35].

In the European IFUs (Instructions for Use) for the Perceval S prosthesis, combined multivalve procedures are not listed among the typical indications for Perceval S implementation. This leads to a limited number of clinical trials and small cohort studies, which mostly arise in experienced high-volume centers. The lack of such studies leads to surgeons’ unfounded hesitation to use this promising technology and does not allow for larger prospective clinical trials.

## 5. Conclusions

Overall, sutureless aortic valve prostheses provide a safe and feasible treatment option in patients undergoing a combined aortic and mitral valve replacement. Compared to the matched conventional surgical group, SU-AVR has proven to be a reliable alternative in patients with multivalve disease, offering shorter procedural (and potentially CPB- and cross-clamp) times and excellent hemodynamic performance. Further evidence as well as randomized control trials on larger cohorts are needed to further assess the outcomes, but with the correct use and cautious sizing, SU-AVR has surely earned its place in multivalve cardiac surgery.

### Study Limitations

This study has some limitations, being retrospective and non-randomized in design, coming from only one center and having a limited number of patients with unequal follow-up periods between the two groups. This may influence the results and reduce the study’s power and increase the possibility of bias. Previous research on this topic has only been conducted on small single-center groups, further larger-scale prospective studies are necessary to validate the method’s safety and effectiveness.

## Figures and Tables

**Table 1:** Baseline characteristics.

Characteristics	Group 1, n = 46	Group 2, n = 23	*p*-Value
Age	71.5 (IQR 54.5–76.0)	74.0 (IQR 68.0–79.0)	0.43
Female sex	25 (54.3)	15 (65.2)	0.39
BMI, kg/qm	26.1 (IQR 24.8–29.9)	25.0 (IQR 23.8–28.9)	0.24
Elective	31 (67.4)	16 (69.6)	0.85
Urgent	10 (21.7)	6 (26.1)	0.69
Emergent	5 (10.9)	1 (4.3)	0.65
Redo	8 (17.4)	4 (17.4)	1
Endocarditis	10 (21.7)	5 (21.7)	1
Arterial hypertension	38 (82.6)	20 (87.0)	0.74
sPAP < 35 mmHg	10 (21.7)	7 (30.4)	0.43
Hyperlipidemia	25 (54.3)	14 (60.9)	0.6
Diabetes	10 (21.7)	4 (17.4)	0.76
COPD	8 (17.4)	6 (26.1)	0.4
History of stroke	5 (10.9)	2 (8.7)	0.78
History of TIA	1 (2.2)	2 (8.7)	0.25
CAD	16 (34.8)	7 (30.4)	0.72
History of PCI	7 (15.2)	2 (8.7)	0.71
Myocardial infarction < 90 days	6 (13)	3 (13)	1
Atrial fibrillation	20 (43.5)	8 (34.8)	0.49
Paroxismal	12 (26.1)	7 (30.4)	0.7
Permanent	8 (17.4)	1 (4.3)	0.25
ICD	3 (6.5)	2 (8.7)	1
Kidney function impairment	8 (17.4)	2 (8.7)	0.4
Creatinine, g/dL	0.9 (IQR 0.77–1.1)	0.95 (IQR 0.74–1.25)	0.6
AR > II	18 (39.1)	9 (39.1)	1
AS	29 (63.0)	16 (69.6)	0.6
AS I–II	6 (13)	2 (8.7)	0.71
AS III	23 (50)	14 (60.9)	0.57
MS > II	19 (41.3)	11 (47.8)	0.61
MR	34 (73.9)	22 (95.7)	0.05
MR I–II	10 (21.7)	7 (30.4)	0.43
MR III	27 (58.7)	15 (65.2)	0.6
TR I-II	8 (17.4)	2 (8.7)	0.3
TR III	12 (26.1)	5 (21.7)	0.69
EuroECORE II	5.8 (IQR 3.3–17.6)	6.0 (IQR 3.3–9.4)	0.95
EF, %	55.5 (IQR 45.7–59.5)	55.0 (IQR 45.0–59.0)	0.64
TAPSE, mm	20.5 (IQR 17.0–23.0)	18.0 (IQR 17.0–21.0)	0.09

AR—aortic regurgitation, AS—aortic stenosis, BMI—body mass index, CAD—coronary arterial disease, COPD—chronic obstructive pulmonary disease, EF—ejection fraction, ICD—implantable cardiac defibrillator, MI—myocardial infarction, MR—mitral regurgitation, PAP—pulmonary arterial pressure, PCI—percutaneous coronary intervention, TIA—transient ischemic attack, TR—tricuspid regurgitation.

**Table 2:** Intraoperative characteristics.

Characteristics	Group 1, n = 46	Group 2, n = 23	*p*-Value
Mechanical MV	2 (4.3)	0	0.55
Perceval S	0	23 (100)	-
S	0	6 (26.1)	-
M	0	10 (43.5)	-
L	0	6 (26.1)	-
XL	0	1 (4.3)	-
Operating time, min	190 (IQR 175.0–230.0)	170.0 (IQR 127.5–232.5)	0.03
CPB time, min	140.0 (IQR 116.2–170.8)	125.0 (IQR 91.5–164.0)	0.06
Cross-clamp time, min	101.5 (IQR 90.5–118.8)	92.0 (IQR 65.0–125.0)	0.11
Intraop blood transfusion, U	2 (IQR 2–4)	3 (IQR 2–4)	0.67
Concomitant procedure	20 (43.5)	10 (43.5)	1
CABG	8 (17.4)	4 (17.4)	1
LAA closure	2 (4.3)	0	0.54
TV repair	14 (30.4)	7 (30.4)	1
ECLS	3 (6.5)	1 (4.3)	0.71
Impella	0	0	1
postop MV-MPG, mmHg	4 (IQR 3.0–4.0)	4 (IQR 3.0–4.0)	0.67
postop AV-MPG, mmHg	6.0 (IQR 5.0–6.0)	6.0 (IQR 6.0–7.0)	0.6
Ventilation time, days	1.0 (IQR 0.25–2.0)	1.0 (IQR 0–4.5)	0.99
ICU stay, days	3.0 (IQR 2.0–5.75)	4.0 (IQR 2.0–10.0)	0.38
Hospital stay, days	10.0 (IQR 8.25–14.0)	8.0 (IQR 7.0–15.0)	0.2

AV—aortic valve, CABG—coronary arterial bypass grafting, CPB—cardiopulmonary bypass, ECLS—extracorporeal life support, ICU—intensive care unit, LAA—left atrial appendage, MPG—mean pressure gradient, MV—mitral valve.

**Table 3:** Postoperative outcomes.

Characteristics	Group 1, n = 46	Group 2, n = 23	*p*-Value
Paravalvular leakage	2 (4.3)	0	0.55
MR > I	1 (2.2)	0	0.47
AR trace	0	0	1
AR relevant	0	0	1
Re-thoracotomy	8 (17.4)	1 (4.3)	0.25
AV-dislocation	0	0	-
LVOT occlusion	0	0	-
Stroke	0	0	-
Permanent pacemaker	0	1 (4.3)	0.33
New onset dialysis	11 (23.9)	4 (17.4)	0.53
Re-intubation	6 (13)	3 (13)	1
Tracheostomy	1 (2.2)	1 (4.3)	0.61
Myocardial infarction	0	0	1
Low output syndrome	6 (13)	2 (8.7)	0.6
RV failure	2 (4.3)	1 (4.3)	1

AR—aortic regurgitation, AV—aortic valve, ICU—intensive care unit, LOS—length of stay, LVOT—left-ventricular outflow tract, MPG—mean pressure gradient, MR—mitral regurgitation, MV—mitral valve, RV—right ventricle.

**Table 4:** Follow-up data.

Characteristics	Group 1, n = 46	Group 2, n = 23	*p*-Value
FU time, days	684.5 (IQR 379.5–1525.8)	234.0 (IQR 34.0–652.0)	0.009
30-day mortality	17%	22%	0.42
6-month mortality	17%	31%	0.4
1-year mortality	22%	31%	0.42
Re-operation	1 (2.2)	0	0.47
Prosthesis endocarditis	2 (4.3)	0	0.55
MPG at FU			
AV-MPG, mmHg	5.0 (IQR 4.0–6.75)	6.0 (IQR 0–6.0)	0.55
MV-MPG, mmHg	3.5 (IQR 2.0–4-0)	4.0 (IQR 0–4.0)	0.8

AV—aortic valve, FU—follow-up, MPG—mean pressure gradient, MV—mitral valve.

## Data Availability

Data are available on request from the authors. The data that support the findings of this study are available from the corresponding author upon reasonable request (E-mail: alina.zubarevich@gmail.com).
